# Distributed Multi-Level Supervision to Effectively Monitor the Operations of a Fleet of Autonomous Vehicles in Agricultural Tasks

**DOI:** 10.3390/s150305402

**Published:** 2015-03-05

**Authors:** Jesús Conesa-Muñoz, Mariano Gonzalez-de-Soto, Pablo Gonzalez-de-Santos, Angela Ribeiro

**Affiliations:** Centre for Automation and Robotics, (CSIC-UPM), Arganda del Rey, 28500 Madrid, Spain; E-Mails: jesus.conesa@csic.es (J.C.-M.); marianogds@hotmail.com (M.G.S.); pablo.gonzalez@csic.es (P.G.-S.)

**Keywords:** supervision system, fault detection, fault recovery, distributed multi-level architecture, autonomous agricultural vehicle, fleet of robots

## Abstract

This paper describes a supervisor system for monitoring the operation of automated agricultural vehicles. The system analyses all of the information provided by the sensors and subsystems on the vehicles in real time and notifies the user when a failure or potentially dangerous situation is detected. In some situations, it is even able to execute a neutralising protocol to remedy the failure. The system is based on a distributed and multi-level architecture that divides the supervision into different subsystems, allowing for better management of the detection and repair of failures. The proposed supervision system was developed to perform well in several scenarios, such as spraying canopy treatments against insects and diseases and selective weed treatments, by either spraying herbicide or burning pests with a mechanical-thermal actuator. Results are presented for selective weed treatment by the spraying of herbicide. The system successfully supervised the task; it detected failures such as service disruptions, incorrect working speeds, incorrect implement states, and potential collisions. Moreover, the system was able to prevent collisions between vehicles by taking action to avoid intersecting trajectories. The results show that the proposed system is a highly useful tool for managing fleets of autonomous vehicles. In particular, it can be used to manage agricultural vehicles during treatment operations.

## 1. Introduction

The use of robotic agricultural machinery to automate agricultural tasks is close to becoming a reality. Automation is a promising step forward that can increase farming productivity due to the potentially high performance of these platforms relative to human labour. For this reason, there has been a significant amount of research in this field in recent years. For example, many advances have been made to achieve autonomous navigation in agricultural vehicles, mainly by using GPS [1,2,3] and computer vision [4,5]. Implements (actuation systems) have also been automated to work selectively and more efficiently. For example, spraying bars integrated with RTK-GPS can now spray selectively with high precision [6], and mechanical-thermal actuators can now burn weeds with high accuracy and correct intensity [7] by detecting the positions and densities of weed patches using cameras installed on the vehicles (ground perception systems) [8,9,10] or using cameras installed on aerial vehicles (remote perception systems) [11,12,13,14]. One important step forward in agricultural automation has been carried out by the RHEA project [15,16,17]. RHEA developed new ways to use automatic systems in agriculture and forestry by proposing a fleet of small, heterogeneous and cooperative robots (unmanned ground vehicles, UGVs, and unmanned aerial vehicles, UAVs) equipped with advanced perception systems, improved implements and enhanced decision-making algorithms to effectively accomplish agricultural tasks, such as chemical and physical control of weeds in crops or pesticide application in woody crops.

Once a task can be automatically executed, it is extremely important to determine whether it does not advance as it was planned as soon as possible and then to accurately identify the causes of the failure. Much of the machinery used in agriculture is heavy and mobile, and therefore, performing a task without human staff and delegating the responsibility to machines is an operation that may be extremely dangerous. In addition, the inherent uncertainty related to a changing and partially unpredictable working environment (e.g., weather, people working nearby, uneven ground, animals that can appear suddenly) makes it more difficult to maintain safe conditions for equipment, people and the crop itself. Consequently, setting up automation to provide the necessary quality is a complex task.

Saffiotti [18] identifies three different strategies to reduce the effects of uncertainty on autonomous systems: (1) eliminating uncertainty; (2) reasoning about uncertainty; and (3) tolerating uncertainty. Some of the uncertainty can be eliminated by using better hardware, such as high-precision machines and sophisticated sensors; however, this strategy increases the cost of the platforms. Engineering the environment can also reduce the uncertainty. For example, artificial landmarks or fixed tracks in the ground can be used. However, this solution reduces both the flexibility and robustness of the agricultural vehicles (for example, the landmarks could be hidden by obstacles). Moreover, there are some sources of uncertainty that cannot be eliminated by engineering the environment. For example, human actions cannot always be predicted. Therefore, reasoning appears to be the best option to address uncertainty; however, it requires more complex models that are not always easy to obtain; in some cases, it is even impossible to obtain these models. Nevertheless, reasoning does not necessarily increase the robustness of the execution because no amount of reasoning can obtain missing information. For example, a robot can guess what is behind a closed door but cannot truly know before the door is opened. Therefore, the only useful strategy to act robustly in a partially unknown and dynamic world is to tolerate uncertainty, *i.e.*, an autonomous platform must be prepared to handle problems during the execution of a task. This approach leads to a type of supervisor system that is able to monitor the execution of a task, control the effects of uncertainty and report to the operator in charge when something is not working as expected.

The majority of such supervisor systems have two main functionalities: fault detection and fault diagnosis [19]. The first functionality detects when something goes wrong, and the second functionality classifies what is going wrong and assesses the magnitude of the fault. Chiang *et al.* [20] list three types of monitoring approaches for these systems: analytical, data-driven and knowledge-based approaches.

Analytical approaches use mathematical models of the system and have two main stages. In the first stage, the measurable inputs and outputs of the system are compared with a model that describes the relationship between the system variables in exact mathematical terms. Any inconsistency in this relationship will indicate a fault in the system. In the second stage, whether a fault has occurred is determined by examining the inconsistencies detected. Analytical approaches are preferable when the system to be monitored is well understood and the uncertainty is limited. However, mathematical models of the system are not always available; in many complex systems, it is difficult or impossible to obtain them. This is the main weakness of the approach, which is already implemented in several supervisors that have been developed for autonomous mobile platforms [21,22,23].

In contrast, data-driven approaches do not require an analytical model of the system, and the information used for fault detection and diagnosis is derived directly from the input data. The decision-making process is often based on statistical methods. Modern industrial systems (entire industrial plants) and autonomous robotic systems are large-scale systems, with heavy instrumentation that produces an extremely large amount of data. Data-driven approaches [24,25,26] have the ability to transform high-dimensional data into lower-dimension space while preserving the essential information. By computing statistical measures, the supervision can be improved significantly in large-scale systems; however, the performance depends greatly on the quality and amount of the input data. Some examples of mobile robot supervisors based on data-driven approaches can be found in the literature [25,26].

Finally, the aim of a knowledge-based strategy is to simulate the behaviour of an expert when solving problems and tasks. In a supervising context, the main opportunity of using knowledge-based approaches is to have the capacity to build hybrid supervision systems by combining analytical and data-driven approaches. As in the other strategies, some examples for unmanned vehicles can be found in the literature [27,28].

This paper describes a complex supervisor system developed to monitor a fleet of automated vehicles performing agricultural tasks. In some cases, the proposed supervisor responds to the analytical approach explained above, whereas in other cases, it is clearly based on a knowledge-based approach. The system is based on a multi-level architecture with multiple supervisors working in parallel and on different supervision levels. A first level is contained inside the equipment on the vehicles and is used to address critical and urgent failures, whereas a second and higher level covers the less pressing failures and addresses unexpected situations that involve several vehicles at the same time, e.g., a collision between two or more vehicles.

In addition to fault detection and fault diagnosis, the system proposed in this paper integrates a third functionality: fault recovery to repair some of the faults detected. The system was tested within the RHEA project scenarios by performing real agricultural tasks. The tasks were supervised for several tests, and the system was able to detect various irregular and dangerous situations, such as tractor out-of-track positions, inappropriate working speeds, incorrect states, malfunctioning implements, unopened spraying nozzles, and malfunctioning sensors on the vehicle. The supervisor was also able to solve some of the problems by using the integrated fault recovery module.

## 2. The Proposed Supervision Approach

First, it is important to define several concepts, such as mission, alarm and supervisor, to understand the proposed approach. The mission is the agricultural task that a fleet must carry out, and it is mainly composed of a plan with the expected trajectories for each unit, the speeds of each unit and the state that the implements must have in each point of the trajectories. For example, in a spraying bar case, the implement may need to be activated or deactivated, or a nozzle of the bar may need to be opened or closed. The alarm is the notification generated when a failure is detected, such as an inappropriate speed, a vehicle out of track or an incorrect nozzle state. For example, in the higher supervision level, in addition to failures, an alarm can also signal important events, such as the accomplishment of a trajectory or the successful initiation of a device. Throughout the document, most of the alarms explained are related to failures, and those that do not refer to malfunctions are explicitly indicated. Finally, a supervisor is the module or piece of code that analyses the information periodically received from the items to be supervised (concrete devices, such as engines, tanks, nozzles, and sensors, or conceptual elements, such as trajectories and collisions), generating an alarm if a failure or important event is detected. In the proposed approach, a supervisor mainly consists of a set of IF-THEN rules that generate alarms when the information collected meets certain conditions. In general, the inputs of a supervisor can be expressed as a pair (property, value), where property indicates the entity to be supervised and value indicates its current state. Supervisors produce several types of alarms, but in general, supervisors in the higher levels generate more types of alarms than those at lower levels because they supervise more complex components and have to consider a more diverse set of failures and important events.

The proposed supervision architecture is distributed over different physical subsystems, taking advantage of the distributed nature of a fleet of tractors working together in agricultural tasks. The supervision can be performed inside the units themselves or can be carried out in an external computer that monitors the entire fleet and is accessible to the operator. In other words, supervision is separated into different levels, which are explained below.

### 2.1. Supervision Levels

**Figure 1:**
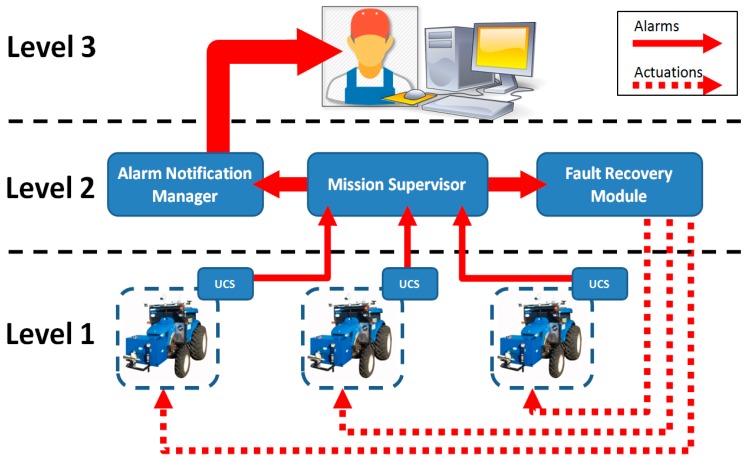
Distributed multi-level supervision.

The first level of supervision includes all of the elemental supervisors running on computers installed in the vehicles. In other words, each elemental supervisor is part of the Unit Control System (UCS) of each vehicle (see Figure 1). At this level, supervisors generate alarms when they detect faults on the onboard subsystems or directly receive alarms from them. Alarms that contain identification codes are generated when specific faulty situations are detected. In some cases, the faults can be solved by the subsystem or by the supervisor on the vehicle. The alarms are always sent to the upper levels, even when the detected fault is repaired inside the vehicle. This procedure is necessary because alarms with a low risk can occasionally be significant if they are combined with other alarms. Therefore, they have to be raised to upper levels to be analysed from the perspective of the entire system.

Supervisors in the mobile units can detect, diagnose and repair a fault. Thus, the first level of supervision performs the three main supervision functions. In addition to the alarm, the ground units send periodic monitoring messages to the second level, external to them, reporting about the status of the vehicle. In the proposed architecture, the second level is divided into three main modules: (1) the Mission Supervisor; (2) the Fault Recovery Module; and (3) the Alarm Notification Manager. The Mission Supervisor processes all of the information provided by the tractors, including both the alarms and monitoring messages, during the execution of the agricultural task (mission), and it detects and diagnoses more complex faults that involve more than one unit, more than one alarm, or unexpected vehicle behaviour. This module also propagates the old and new alarms to the Fault Recovery Module and Alarm Notification Manager. The Fault Recovery Module receives the alarms generated by the Mission Supervisor and uses a pre-established protocol to find the action needed to address the fault. At this level, the Mission Supervisor performs fault detection and fault diagnosis, and the Fault Recovery Module performs fault recovery. Finally, the Alarm Notification Manager is a policy system that decides whether an alarm needs to be sent to the third level of supervision. This decision is based on a set of policies that consider the priority and severity of the alarm.

The third level, the operator level, is related to the graphical user interface (GUI). It is used to convey information to the operator in charge of mission supervision. With the approach proposed, the operator receives a sufficient amount of information, generated in the lower levels, to keep track of mission performance. The operator is the final decision element of the proposed supervision architecture. In fact, if something does not work as expected, the operator can take control of the fleet and directly change the guidelines of mission execution. Thus, even at this level, the three main supervision functionalities, *i.e.*, fault detection, fault diagnosis and fault recovery, can be carried out. The Mission Supervisor, as well as the Fault Recovery Module and Alarm Notification Manager, are explained in detail in Section 2.2, Section 2.3 and Section 2.4.

### 2.2. Mission Supervisor

**Figure 2:**
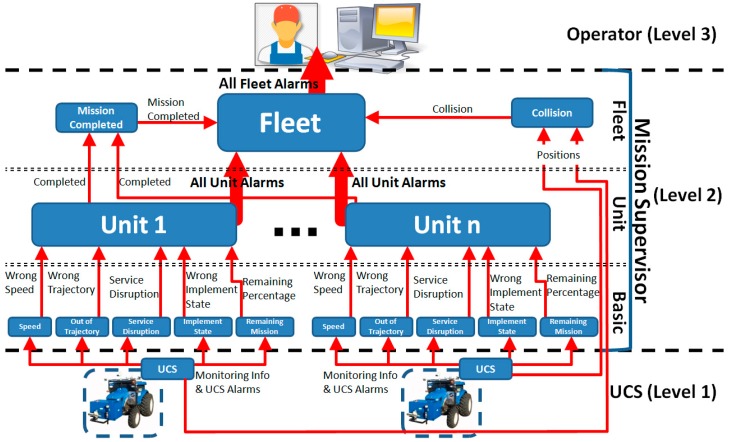
Supervision architecture. The rounded rectangles represent supervisors at different levels, and the arrows represent their inputs and outputs, alarms and monitoring information. The information is provided by the supervisors that are installed on the tractors.

In the proposed approach (see Figure 2), the Mission Supervisor consists of a set of individual supervisors working together to manage different items.

For example, one supervisor monitors the speed of the unit, another supervisor monitors the trajectory, and so on. In this manner, the mission supervision is distributed across different levels: basic, unit and fleet levels. Thus, it is possible to execute only certain supervisors (if desired) or to update one of them easily without affecting the others. Moreover, by dividing and properly combining individual supervisors, complex behaviour can emerge from the Mission Supervisor, as will be described later. The supervisors of the basic level receive only the information associated with a specific property that can be related to a physical entity, such as a nozzle or sensor, or a conceptual item, such as a trajectory. Consequently, the property supervisors contain only the logic to detect the failures related to their associated properties. At the unit level, supervisors detect higher-level failures that arise from different properties of the same unit. For example, when more than one nozzle does not work, they can detect that situation. Finally, at the fleet level, supervisors detect anomalies pertaining to the behaviour of the entire fleet, such as a collision of several units.

In addition to the information provided by vehicles, the supervisors can also access mission data, such as the defined trajectories for each of the units, their speeds, and the states of the implements. This information is static; it does not change during mission execution. It is established for each supervisor at the beginning of the mission.

The internal logic of the supervisor modules encapsulates the fault detection functionality required for any supervision system. The fault diagnosis functionality is encapsulated in the alarms because the types of alarms issued by the supervisors determine the types of failures.

With this approach, the supervision behaviour is clearly decoupled because each supervisor encapsulates part of the logic of the supervision and the supervisors can all be easily replaced. Moreover, the approach is also hierarchical because it allows the supervisors to link to each other and build more complex supervisors that perform supervision at different levels. By combining decoupling and hierarchy, any supervision system built with this approach can be easily adapted and updated to provide new supervision functions.

### 2.3. Fault Recovery Module

The Fault Recovery Module is the module in charge of neutralising the failures reported by the alarms. Thus, this module encapsulates the fault recovery functionality. It receives the alarms issued by the Mission Supervisor and refers to a database to determine the strategy that must be executed to counteract the alarm. The strategy consists of a set of actions to be executed by the UCS. For example, if a collision of several units is predicted, the neutralising strategy may be to stop the units to avoid the collision. In general, the strategies can involve one or more actions that must be executed by the UCS. Such actions may include reducing the speed, restarting the mission, and changing the pressure of a nozzle.

### 2.4. Alarm Notification Manager

The Alarm Notification Manager is a policy system that decides which of the received alarms should be sent to the operator (level 3) according to a set of policies. This allows alarms to be filtered in certain situations. As an example, consider the case of a “pilot flame” alarm in a mechanical-thermal tool. Such tools are able to use flames to remove weeds from fields [7]. In this type of implement, the pilot flame may become extinguished many times (due to wind or other causes). The fault can be detected and repaired by the onboard actuation system of the tractor, which is able to reignite the pilot flame repeatedly. However, it is possible that a larger problem will occur that only can be detected at higher levels of supervision, such as the Mission Supervisor. This may occur when the alarm is permanently active, for example. In this case, a revision of the tool operation is required, and therefore, the farmer/operator must be notified (level 3) by an alarm generated at level 2 related to the larger problem. The operator need not be notified of the minor alarms generated by the onboard actuation system of the vehicle. This strategy helps to generate alarms in a timely manner without overwhelming the operator with excessive, distracting messages. For completeness, a log of all alarms generated during the mission is stored so that the operator can review it after the mission as needed.

## 3. Characteristics of the Fleet to Be Supervised

**Figure 3:**
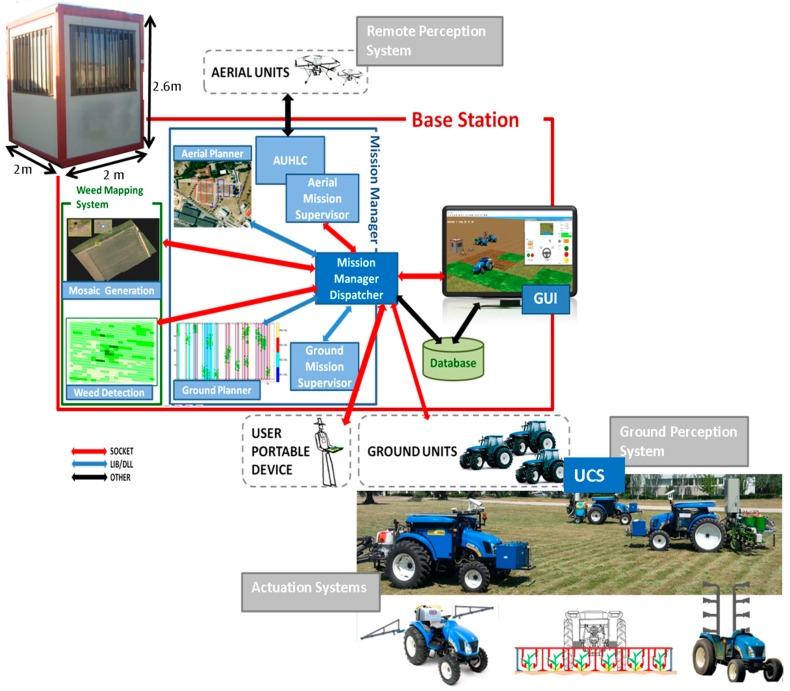
RHEA architecture.

A fleet of vehicles with the architecture of the RHEA European project [29] was used to test the proposed supervision approach. Figure 3 shows the RHEA architecture. Aside from the vehicles, there is a base station that contains a main computer to remotely manage the fleet and execute other agricultural services, such as generating a weed map from aerial images taken by UAVs or generating the plan that the ground units will use to move during the mission. This computer executes the supervision levels external to the UCS, that is, levels 2 and 3 (Figure 1). It is a desktop computer with motherboard ASUS Z87-K SK1150/PCX 3.0, Intel core i7 4771 3.5 GHZ CPU, 2 DDR3 1600 8 GB PC3-12800 modules (16 GB RAM), and SSD with 240 GB. The power of these features was sufficient for operating the Mission Supervisor, the Fault Recovery Module, the Alarm Notification Manager and additional software, such as the operator GUI to display the supervision outputs.

**Figure 4:**
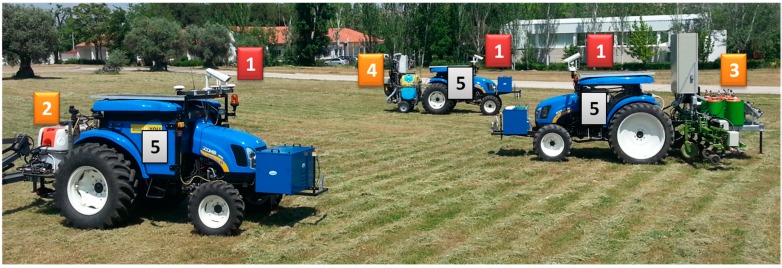
RHEA fleet: (1) Perception systems; (2) sprayer boom; (3) mechanical-thermal tool; (4) air-blast sprayer; and (5) unit controller.

The RHEA fleet contains three autonomous tractors that can be configured to use different implements. Each vehicle is based on a restructured medium New Holland Boomer 3050 (50 hp, 1270 kg), in which the cabin has been reduced to leave just sufficient room for the computer equipment required for the perception, actuation, location, communication, and safety systems. Other equipment has been integrated outside of the cabin, such as an RGB camera, a laser, three antennas (two for the GPS receiver and one for communications), emergency lights and bottoms, a fuel cell and a solar panel placed on top of the vehicle. Figure 4 shows the three units that were developed, as well as the perception systems and implements.

In the following sections, the different types of equipment on the tractors are presented, with special attention to the information needed by the supervisor system to monitor the performance of the units during the mission.

### 3.1. Perception Equipment

Each vehicle in the fleet is equipped with several sensors to detect changes in the environment. The details have been previously described [30]; however, a brief summary is given below. The vehicles have four main sensors: an Inertial Measurement Unit (IMU), a camera, an RTK-GPS receiver and a Light Detection and Ranging (LiDAR) system.

The IMU is a 3DM-GX3^®^-35 high-performance model miniature Attitude Heading Reference System (AHRS) developed by LORD MicroStrain^®^ Sensing Systems (Cary, NC, USA), that provides the pitch and roll angles of the vehicles. The camera is a SVS4050CFLGEA model from SVS-VISTEK (Seefeld, Germany) and is built with the CCD Kodak KAI 04050M/C sensor with a GR Bayer colour filter; it provides high-resolution images (2336 by 1752 pixels with a 5.5 by 5.5 μm pixel size) to accurately determine the locations of the weeds and crop lines. Because the agricultural tasks are typically carried out under bad weather conditions that can damage electronics, the camera and IMU are placed inside a housing unit with a fan controlled by a thermostat for cooling purposes. This allows them to work even when it is raining or when the temperature is above 50 °C. Both devices are also sufficiently robust to continue working in real time even in the face of vibrations produced by the vehicles when moving through the field.

The RTK-GPS receiver on each tractor is a Trimble BX982 GNSS receiver; a multi-channel, multi-frequency OEM GNSS receiver that enables OEM and system integrators to rapidly integrate centimetre-level positioning and precise headings into their applications. Moreover, a dual-antenna input system is also integrated to accurately determine the heading of the vehicles; this system exceeds the performance of a single-antenna GNSS system. A single-antenna system would have difficulty determining where the antenna is positioned relative to the vehicle and object of interest, especially when the dynamics are low. Thus, the BX982 supports two antennas connected to the board by using dual chips. The independent observations from both antennas are passed to the processor, where multi-constellation RTK baselines are computed and compared with the positions provided by both antennas. As the real physical distance and their positions on the vehicle are known, it is possible to calculate the vehicle’s heading with high accuracy. Therefore, a single connection to the tractor receiver via RS232, USB, Ethernet or CAN delivers both centimetre-accuracy positions and a heading that is accurate to less than a tenth of a degree (2 m baseline). In this manner, both the position and heading of the vehicles are provided with high precision. The RTK-GPS receivers can report the vehicle’s current position with a relative accuracy of 0.008 + 1 ppm (parts per million) in the horizontal plane, which is always below 2 cm, and a maximum frequency of 20 Hz.

Finally, the vehicles are also equipped with a LiDAR sensor to detect obstacles as an additional security safety system in case the supervisor system does not detect them. The LiDAR sensor used was model LMS 111 (SICK AG, Waldkirch, Germany). It was installed on the middle of the vehicle’s front with a push–broom configuration (4° inclination) and was used to detect obstacles along the vehicle trajectory with a ground clearance of 70 cm).

### 3.2. Implements

An implement is a device designed to perform an action on a crop. In a precision agriculture scheme, some elements on the implements can be operated independently, and PLCs and computers are used to control them and coordinate their actions with the vehicle. To date, in the RHEA project, the following three implements have been developed: a sprayer boom, a mechanical-thermal tool and an air-blast sprayer.

The herbicide tool is integrated [31] by a 6-m spray boom that is divided into 12 sections, which can be independently activated, and 2 tanks. The main tank has a capacity of 200 L, and the secondary tank has a Direct Injection System (DIS) that allows the chemicals to be injected directly at the end of the bar, avoiding mixing of the herbicides with water in the main tank before the treatment application. With the DIS, it is possible to recover excess herbicide, which provides important environmental and economic advantages. Both tanks are supplied with optical sensors that check the liquid volume in the tank.

The mechanical-thermal equipment is devoted to weed control in flame-resistant crops, such as maize, onion, and garlic. The implement consists of a mechanical tool for removing weeds that have grown in the inter-row space and a burn system to perform selective and accurate burning in the rows [7,32]. The mechanical actions are continuously performed, independent of the presence of weeds; in contrast, the thermal treatment is performed only for the weeds that appear in the crop rows. Thus, the treatment system must be attached to a weed detection system. In the RHEA project, this system has been developed to process the RGB images acquired with a camera on the tractor in real time [30,33,34]. The pressure of the liquefied petroleum gas (LPG, the fuel source for the burners) is adjusted according to weed cover; two levels of pressure have been defined.

The air-blast sprayer for pesticide applications in olive trees consists of two main vertical columns that support two sets of nozzles. The upper and lower nozzles in each column can rotate in the range of 0°–30° to adjust the spray direction to the canopy size. The two central nozzles in each column are static. Each nozzle has an associated ultrasonic sensor that turns on (off) the nozzle if it detects (does not detect) an object in front of the working range of the nozzle. In addition, the upper (lower) nozzles move down (up) if they do not detect an obstacle in the working range [35,36]. This implement was used in the RHEA project to spray olive trees; thus, it is assumed that the potential obstacles are the olive tree canopies.

### 3.3. Other Devices

The tractors are equipped with an onboard computer that runs the UCS. A CompactRIO model 9082 from National Instruments (Austin, TX, USA) was selected due to its high-performance multicore system for intense embedded monitoring and control applications. It has a 1.33 GHz dual-core Intel Core i7 processor, 32 GB of nonvolatile storage and 2 GB of DDR3 800 MHz RAM. It also includes a LX150 FPGA for custom I/O timing, control and processing.

## 4. The Implemented Supervision System

This section describes the main characteristics of the Supervision System (Mission Supervisor, Fault Recovery Module and Operator GUI) implemented for RHEA based on the proposed approach. The outputs of the UCS, *i.e.*, the alarms and monitoring messages, are listed in Table 1 and Table 2, respectively.

**Table 1:** UCS alarms.

Producer System	Alarm Type	Description
Unit Control System (UCS)	Critical mainboard temperature	The system needs to cool down to prevent damage to the communication system hardware
	Critical CPU temperature	The system needs to cool down to prevent damage to the communication system hardware
	Critical RAM storage level	Alarm related to the logging and monitoring functionalities, indicating possible loss of data
	Application traffic logging error	Application traffic logging failed
	Communication synchronisation error (GPS time)	Synchronisation to global GPS time failed. Thus, there is no common knowledge of system time
Vehicle	Mission aborted: Unit not moving	The unit cannot move due to some internal error
	Mission aborted: Unit stopped	The unit was stopped successfully by external request
	Mission finished	The mission sent to the unit was executed successfully
	TPH is not moving	Three point hitch (TPH) is not moving
	Error in set/unset PTO	The power take-off (PTO) value could not be set
	Set/Unset implement error	The implement could not be set
Sprayer boom	Main tank volume critical	The level of the main tank is very low
	DIS tank volume critical	The level of the Direct Injection System (DIS) tank is very low
	Impossible to adjust main flow	The main flow could not be set
	Herbicide line blocked	The herbicide line is blocked
	Boom is opening or closing	Boom is opening or closing
	Impossible to adjust boom opening	The boom could not be opened
	Start/Stop failure	The implement was not started/stopped successfully
	Change nozzles failure	The nozzles could not be set properly
	Open/close Boom failure	The implement was not opened/closed successfully
	Set main flow failure	The main flow value could not be set
	Set DIS flow failure	The Direct Injection System (DIS) flow value could not be set
Flaming tool	Bottle empty	The LPG bottle is empty
	Start/Stop failure	The implement was not started/stopped successfully
	Change burners failure	The burners could not be set properly
Air-blast sprayer	No flow in main line	There is no flow in the main line
	Tank level critical	The level of the pesticide tank is very low
	US sensor not working	The ultrasonic sensor (US) is not providing information
	Start/Stop failure	The implement was not started/stopped successfully

**Table 2:** UCS monitoring information.

Producer System	Message Type	Description
Vehicle	Controller	Indicates the state of the internal controller: e.g., disabled, ready, manual or executing a moving operation, paused, or stopped.
	Motion	Provides the GPS position, the speed, the heading and the PTO and hitch states.
Flaming	Implement	Indicates the state of the implement (ON/OFF)
	Burners	Reports the state of the burner pressure (OFF/Low/High)
Sprayer boom	Implement	Indicates the state of the implement (ON/OFF)
	Nozzles	Reports the state of the valves in the spraying bar (ON/OFF)
Air-blast sprayer	Implement	Indicates the state of the implement (ON/OFF)

The Alarm Notification Manager sends all alarms to the operator except the free path alarms in this version of the Supervision System.

### 4.1. Mission Supervisor

The Basic Level contains the supervisors that directly manage the information provided by each UCS. These supervisors monitor the speed, trajectory, service disruptions, implement states and remaining mission status (see Table 3). In particular, the speed supervisor receives the current speed and position of each vehicle (both provided by the RTK-GPS receiver) and periodically verifies that the received speed matches the expected working speed (within a certain margin of tolerance), which was previously set according to the task’s specifications. An alarm is generated when they do not match. Similarly, the out-of-track supervisor periodically checks whether the current position of the unit matches the expected position based on the mission trajectory, and it produces an alarm whenever a point is missed or is visited out of order. The position accuracy and frequency (2 cm and 20 Hz, respectively) of the RTK-GPS receivers guarantee that a vehicle cannot move far without being detected by the supervisor. The service disruption supervisor periodically checks the most recent time that a sensor or internal system has provided its information. If some critical time threshold is exceeded, a service disruption alarm is issued. The overall system supervises moving medium-size tractors, and thus, it is essential to receive critical information, such as the tractor position, with the appropriate frequency. The implement state supervisor verifies the state of the actuators in the onboard tractor implements and generates an alarm if an unexpected state is found. The remaining mission supervisor generates an informational message whenever it receives the vehicle location (UTM coordinates), and the message includes details about the remaining percentage of the mission. This message cannot be considered an alarm because it does not represent a malfunction in the system. It is useful to notify the operator about the percentage of the mission remaining at any time.

In the current version of the implemented Supervision System, the Unit Level encapsulates all of the supervision associated with a unit by bringing together all of the alarms related to it. Therefore, this level contains as many supervisors as there are units in the fleet. Thus, in the proposed approach, the basic level supervisors are simple. They separately detect minimal deviations, and by working together, they form a powerful tool that covers a wide range of situations.

**Table 3:** Basic-level supervisor alarms.

Supervisor	Alarm	Description
Speed	Wrong Speed	The unit is not moving at the expected speed.
Out of Track	Wrong Position	The unit is not at the expected location.
Service Disruption	Service Disruption	A service (such as a sensor or subsystem) has not provided information within the required time.
Implement State	Wrong Implement State	The implement is not in the expected state (e.g., a nozzle is not opened)
Remaining Mission	Remaining Mission Percentage	The percentage of the mission that remains, which is included in the message as a variable.

Finally, three of the supervisors are contained in the Fleet Level: the collision supervisor, mission completed supervisor and fleet supervisor. They generate the alarms listed in Table 4. The collision supervisor detects potential collisions by analysing the positions of all units in the fleet and their planned trajectories. The supervisor works in two steps: (1) Given a distance threshold, the supervisor checks two by two to determine whether the units are close each other. If the threshold is exceeded, then an alarm is generated. If the threshold is not exceeded, the supervisor looks for the current positions of the units inside the planned trajectories and calculates where they will be in the near future by advancing them incrementally according to the plan. The positions are advanced by small time intervals that guarantee the detection of trajectory intersections and the production of appropriate alarms. An increment of time between 0.5 and 1 s was experimentally determined to be sufficient for the given unit size and the expected maximum speed, which is approximately 6 km/h in herbicide treatment tasks. The security area of the units is defined by considering the real dimensions of the unit and an additional security distance that is dependent on the vehicle speed: a faster unit will have a longer security boundary as the braking time is highly dependent on the vehicle speed. The current positions (and thus their areas) are advanced incrementally until some maximum time. The value of the maximum time was defined to allow sufficient time to perform the following set of actions before a collision actually takes place: detecting the collision, alerting the operator and letting him act, if needed. In addition to the alarm, the collision alarms contain extra information, such as the identification of the involved units and the collision risk level (low, medium or high), which is calculated according to the remaining time to collision: the lower is the amount of remaining time, the higher is the risk.

Because of the criticality of a collision situation, it is essential to link these alarms to neutralising actions that counteract the damage as quickly as possible. The Fault Recovery Module is the piece of the Supervision System devoted to determining the most appropriate action to counter an alarm. In the case of a collision, a possible neutralising action can consist of pausing one of the units involved in the collision. In general, this action is sufficient to avoid a collision. However, there are some special cases in which both units must be paused, such as if they are approaching each other by following the same path in opposite directions. Furthermore, when one or more vehicles have been paused, they should be resumed once the risk has been overcome. Thus, it is necessary to detect when the path is free again for the paused units. The collision supervisor is also in charge of checking the status of the path by following a procedure similar to the one used to detect a collision. The current position of the unit is obtained, and whether the area of the unit will overlap the area of another unit in the near future is determined by incrementally updating the positions of the fleet. If no collision is detected, then an alarm is issued reporting that the path is free. The mission completed supervisor receives the remaining mission alarms propagated from the lower level, and it generates an alarm to report to the operator that the mission is over when all of the remaining mission alarms are zero, *i.e.*, when all units have completed their plans.

**Table 4:** Fleet-level supervisor alarms.

Supervisor	Alarm	Description
Collision	Very Close	Two units are very close
	Collision	Two units are going to collide in the near future
	Free Path	A unit has been paused due to an impending collision but now has a free path to continue.
Mission Completed	Mission Completed	All of the units have completed their missions.
Fleet	ALL	All of the alarms generated at this level and at lower levels.

In summary, the fleet supervisor brings together all of the alarms related to the fleet. It is helpful to encapsulate all of the supervision in a single supervisor. All of the alarms generated by this supervisor are directly connected to the Fault Recovery Module.

All supervisors were developed into C++ using the Qt libraries [37]. The use of C++ guaranteed a fast performance, which is required due to the vast amount of information to be processed; the Qt libraries simplified many of the implementation aspects, such as collision detection (using the Qt graphics scene package), the internal communication (using the signals/slots methodology) among the supervisors and the external communication to other modules (using the Qt socket classes integrated into the Qt network package).

### 4.2. Fault Recovery Module

The Fault Recovery Module can initiate actions in the fleet when it receives certain pre-specified alarms. For this version of the system, only the alarms generated by the supervisors of the highest level, the collision supervisor and the mission completed supervisor, trigger actions. Table 5 summarises the actions associated with these alarms.

**Table 5:** Alarms and actions associated with the Fault Recovery Module.

Alarm	Action
Very Close	Stop the units involved
Collision	Pause the units involved
Free Path	Resume the unit
Mission Completed	Stop all units in the fleet

When the units are extremely close to each other, they are directly stopped to avoid potential damage. If a near-future collision is detected, the situation is not as dangerous as in the first case (there is more reaction time), and thus, a pause is sent. Once the path is free for a paused unit and a free path alarm is received, a resume action is sent to the unit. If a mission completed alarm is received, the Fault Recovery Module sends a stop to all units, ensuring that the fleet finishes properly.

In addition the Mission Supervisor, the Fault Recovery Module was developed using Qt and was connected to the Mission Supervisor by the signals/slots methodology to accelerate the interactions between both modules.

### 4.3. Operator GUI

The operator GUI was developed using the mobile robotics simulation software Webots 8 [38], which is developed using Qt. Thus, the communication between the GUI and the Mission Supervisor modules were guaranteed using Qt sockets.

## 5. Results and Discussion

**Figure 5:**
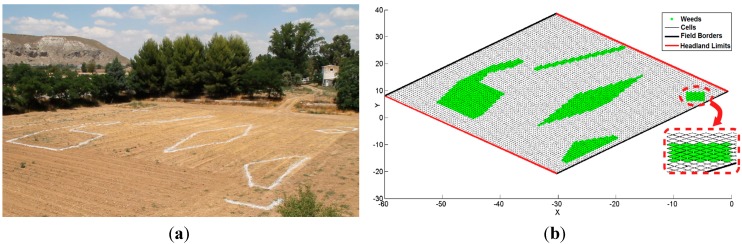
Actual test field and its computer representation. Map of the field and weeds used for the tests. The white markings define the areas of weed patches.

Two different missions were planned to test the supervision system. The first mission was designed to check the general behaviour of the supervision system: a single tractor carrying a sprayer boom had to cover the crop by applying treatment just on the weed patches. The second mission was designed to check the supervision system behaviour when several units are working together, and it was designed to validate the Collision Supervisor and Fault Recovery Module: two tractors had to cover the entire field and avoid collisions. The missions were executed in the facilities of the Center for Automation and Robotics in Madrid (40°18′51.102″N, 3°29′03.379″W). The field was 42.5 m × 41.5 m, and it was manually prepared by painting white lines that delimit the simulated weed patches (Figure 5a). Then, a weed map was built by using an RTK-GPS receiver to accurately position the borders of the weed patches (Figure 5b). Additionally, the four corners of the field were also delimited by white lines. The weed map consisted of a matrix in which each cell represents an area of 0.5 m × 0.5 m of the crop; it takes a value 1 if there is weed and a value of 0 otherwise. The UCS systems were set to issue monitoring messages every 250 ms (4 Hz) for both missions; this frequency was sufficient to control the vehicles if they did not exceed 3 km/h. A vehicle moves approximately 0.83 m each second at 3 km/h, *i.e.*, 21 cm per message, which is a highly controllable distance, as experimentally proven. The missions were executed several times, and the results were similar for all of the attempts.

### 5.1. One Vehicle Mission

**Figure 6:**
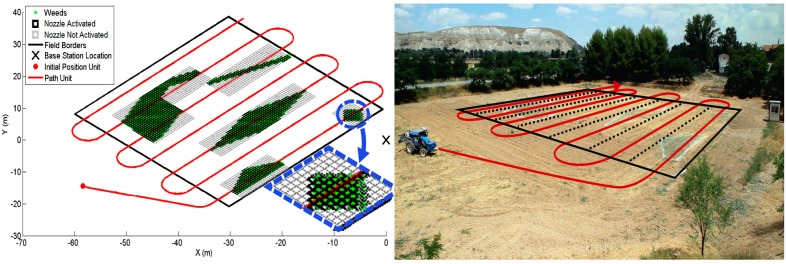
Mission trajectory and sprayer boom activation status.

In this mission, the tractor had to cover the field and apply herbicide only in the appropriate patches. Because the patch positions were well known, the mission trajectory, represented in red in Figure 6, was designed to cover the entire field and account for the 6 m (12 nozzles) bar that was carried by the tractor. On the left side of Figure 6, the black squares show where the nozzles are active, and the grey squares indicate inactive nozzles when at least another nozzle in the bar is activated.

**Figure 7:**
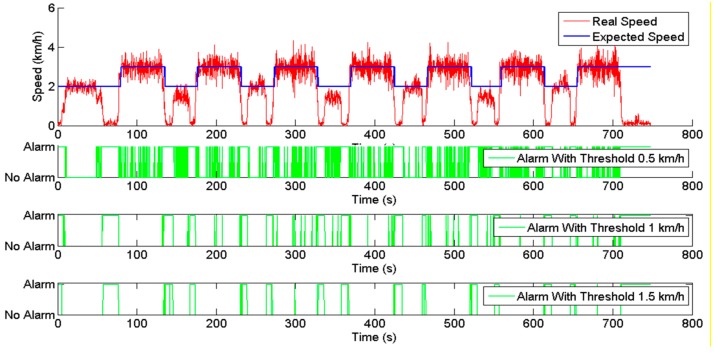
Speed supervisor inputs (real and expected speed) and outputs (alarms).

Figure 7 shows the results obtained for the speed supervisor. The expected working speeds for performing the task were 2 km/h in the headlands (during the turning manoeuvres) and 3 km/h inside the field. The supervisor was tested with three different error thresholds: 0.5, 1 and 1.5 km/h. The actual and expected speeds of the tractor are displayed in red and blue, respectively, at the top of the figure. At the bottom, the output alarms generated for the three different speed thresholds are displayed in green. The supervisor was initially tested with the smallest speed threshold, 0.5 km/h, and many alarms were generated because the vehicle was not able to maintain the requested speeds within such a narrow margin. Then, two higher thresholds were tested, 1 and 1.5 km/h, and the alarms generated for these cases are displayed just below. In general, the speed supervisor was capable of successfully detecting the differences between the actual and expected speeds and generated alarms when the differences were larger than the set threshold.

**Figure 8:**
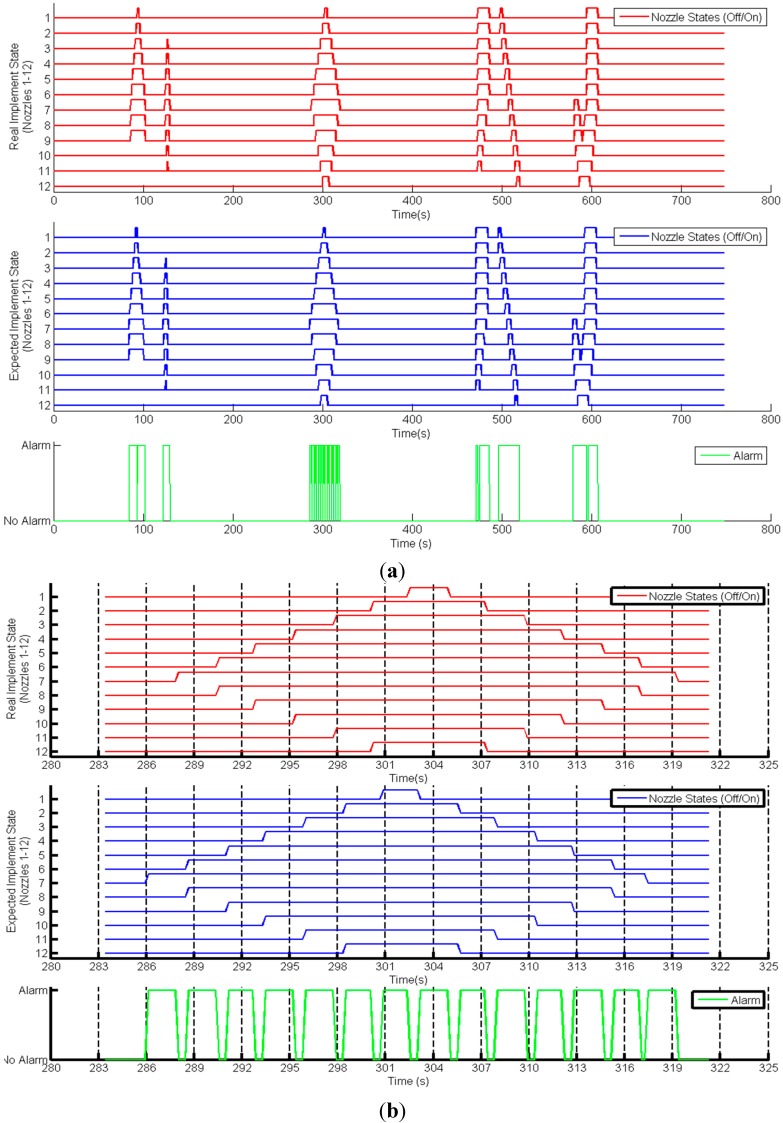
Implement supervisor inputs (actual and expected states) and outputs (alarm) (**a**) in the entire mission; and (**b**) in the mission interval from 283 s to 323 s.

Figure 8 shows the results for the implement state supervisor for the sprayer boom. Figure 8a shows the entire mission, whereas Figure 8b displays only the part of the mission that elapsed from second 283 to second 322. The actual and expected states of the nozzles are shown in red and blue, respectively, and the alarm activations/deactivations are indicated in green. The status differences were mainly due to the delay associated with the response time of the nozzles and were successfully detected by the supervisor. Because the supervisor is checking the implement state with a frequency (every 250 ms) higher than the nozzles’ response time, an alarm is generated every time the UCS has to activate/deactivate the nozzles. These differences are on the order of seconds and occasionally even smaller; thus, distinguishing the differences between the expected states and the actual states in Figure 8a is nearly impossible. The nozzle states precisely matched the shape of the patches. For example, the large diamond inside the third track of the field (refer to Figure 6) can be easily observed near 300 s in both figures.

**Figure 9:**
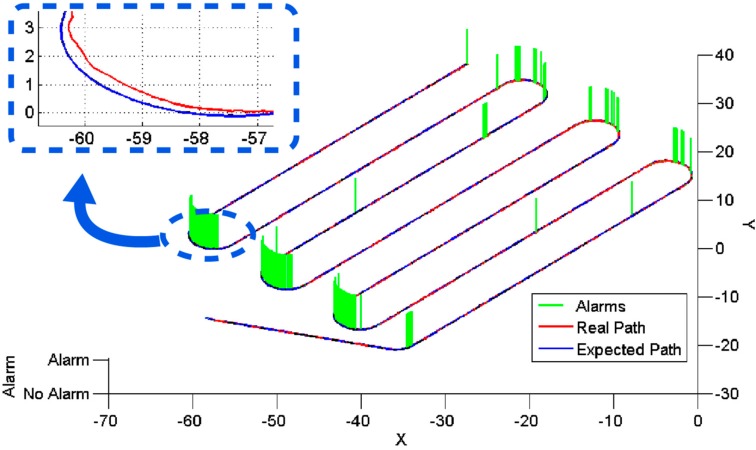
Out-of-track supervisor inputs (actual and expected trajectory) and outputs (alarm).

Figure 9 shows the results for the out-of-track supervisor. A point is set as visited when the distance between the expected and actual positions of the tractor is equal to or less than 10 cm. The actual and expected trajectories are shown along the time in red and blue, respectively, and the alarm activations/deactivations are indicated in green. Most of the alarms are generated during the turning manoeuvres. For the turns, the vehicle accuracy is lower because the wheels are more affected by the roughness of the terrain. Furthermore, the minimum turning radius of the vehicle used was 2.89 m, and the mission path involved manoeuvres with a turning radius of 3 m, and thus, the turning machinery was working under extreme conditions.

**Figure 10:**
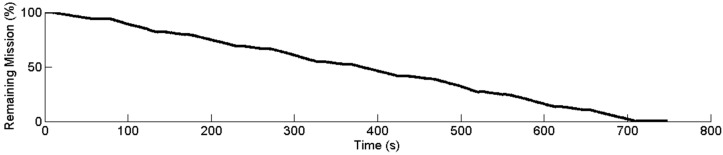
Remaining mission supervisor output.

Figure 10 shows the results for the remaining mission supervisor. As shown in Figure 9, the tractor covered the trajectory quite accurately, and the remaining percentage of the mission can be easily determined by calculating the remaining distance from the current position. The remaining mission supervisor succeeded in updating the remaining percentage periodically; this percentage is shown in Figure 10. Because the turning manoeuvres were accomplished at a lower speed (2 km/h) than the straight lines (3 km/h), the remaining percentage decreased more slowly during the turns; this is why the slope is not constant in Figure 10.

**Figure 11:**
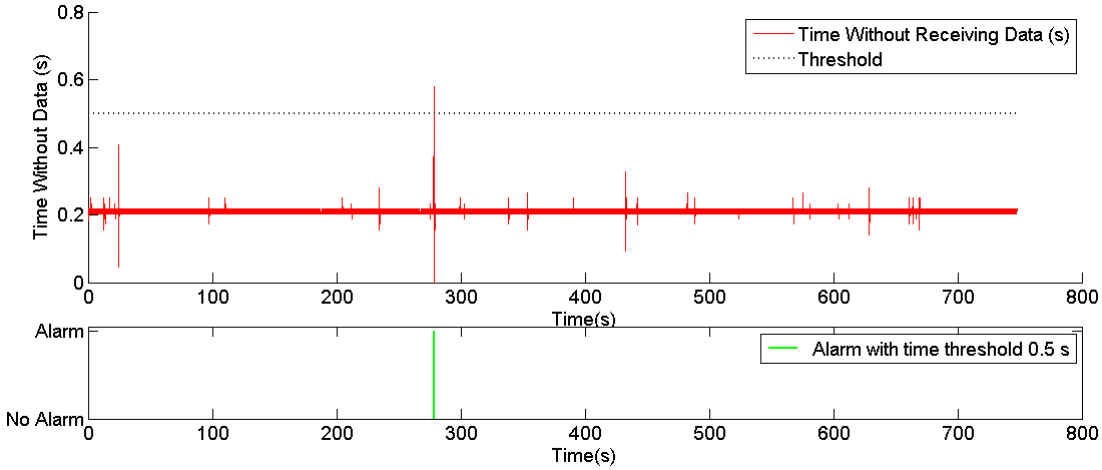
Alarms generated by the service disruption supervisor.

**Figure 12:**
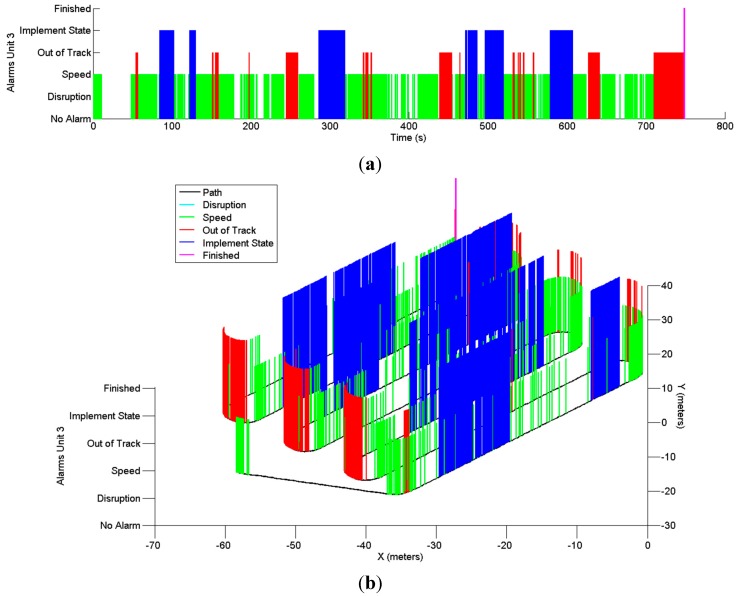
Alarms generated by the unit supervisor (**a**) over time and (**b**) over the trajectory.

Figure 11 shows the results for the service disruption supervisor. This supervisor was set to monitor the positions provided by the GPS receivers on the vehicles. Two time thresholds were tested to verify whether the messages arrived within a given expected time window: 1 s and 0.5 s. As the monitoring rate provided by the UCS was set to 4 Hz, the majority of the monitoring messages were received with an interval time of 250 ms, as shown in the figure. In one case, the position was not received for a period close to 0.6 s. As this period did not exceed the 1 s threshold, no alarm was generated for that time limit. For the remaining time limit of 0.5 s, an alarm was issued near 300 s.

Finally, Figure 12 shows the unit supervisor, which brings together all of the alarms generated for the tested vehicle. The speed alarms displayed were generated when the speed error threshold was set to 0.5 km/h. There are no service disruption alarms because the time limit was set to 1 s for the test.

In this case, there is not a fleet; thus, the fleet supervisor is equivalent to the unit supervisor because the fleet is composed of only one vehicle. A video describing the mission can be accessed in [39].

### 5.2. Fleet Mission

**Figure 13:**
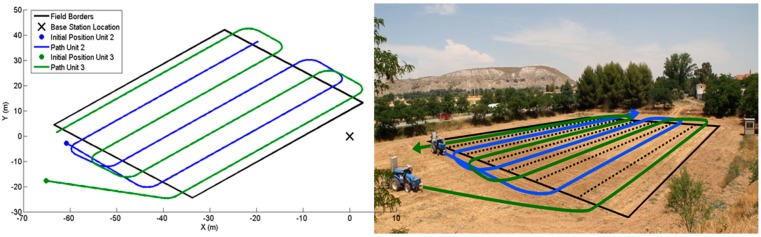
Trajectories for the two tractors in the second mission.

The second mission was focused on testing the supervisor with several units working together; thus, the supervisors that involve several units (specially the collision supervisor) are analysed below. A mission with two vehicles turning on the same headlands at the same time was generated. Figure 13 shows the trajectories of the mission.

**Figure 14:**
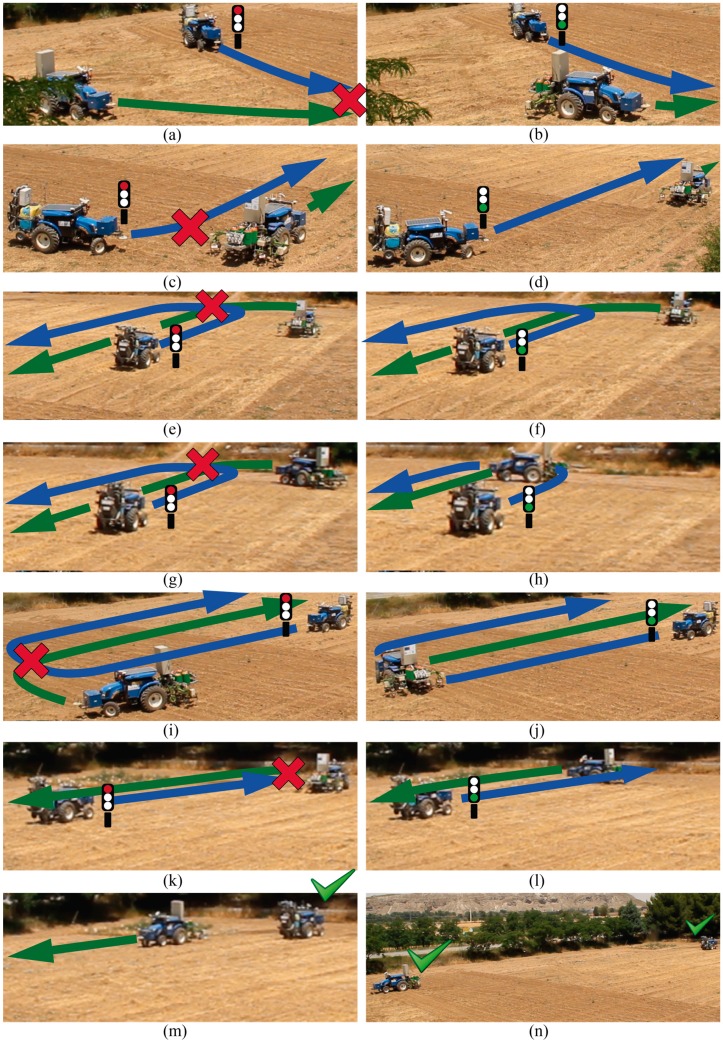
Mission timeline. (**a**–**l**) Collisions dealt with by pause and resume operations, and (**m**–**n**) final positions of the vehicles.

The tractors had to cover the entire field by following parallel trajectories at the same speed, faking a spraying treatment; thus, the supervisor was configured to assume that both tractors were carrying 6 m spraying booms. Furthermore, the trajectories involved potential collisions during the turns on the headlands, as shown in Figure 13 and Figure 14. Figure 13 shows the initial positions, field contours (black lines) and trajectories (blue line for vehicle identifies as 2 and green line for vehicle identifies as 3). Figure 14 displays the most relevant moments of the mission. At the beginning of the mission (Figure 14a), both tractors started moving, but a collision was detected close to the right bottom corner of the field; there, both tractors had to turn, and, even if they had not collided, their bars (6 m) would have collided during the turn. The width of the tracks that divide the field (delimited in Figure 13 by the black dotted lines) is equivalent to the bar length. Moreover, the collision supervisor detects the collisions by calculating where the units will be over the mission time, but it considers a surrounding area larger than the physical one for security reasons because the tractors cannot be stopped instantaneously. Thus, the supervisor will detect a collision even if there is no real collision but the units move inside this safety area.

**Figure 15:**
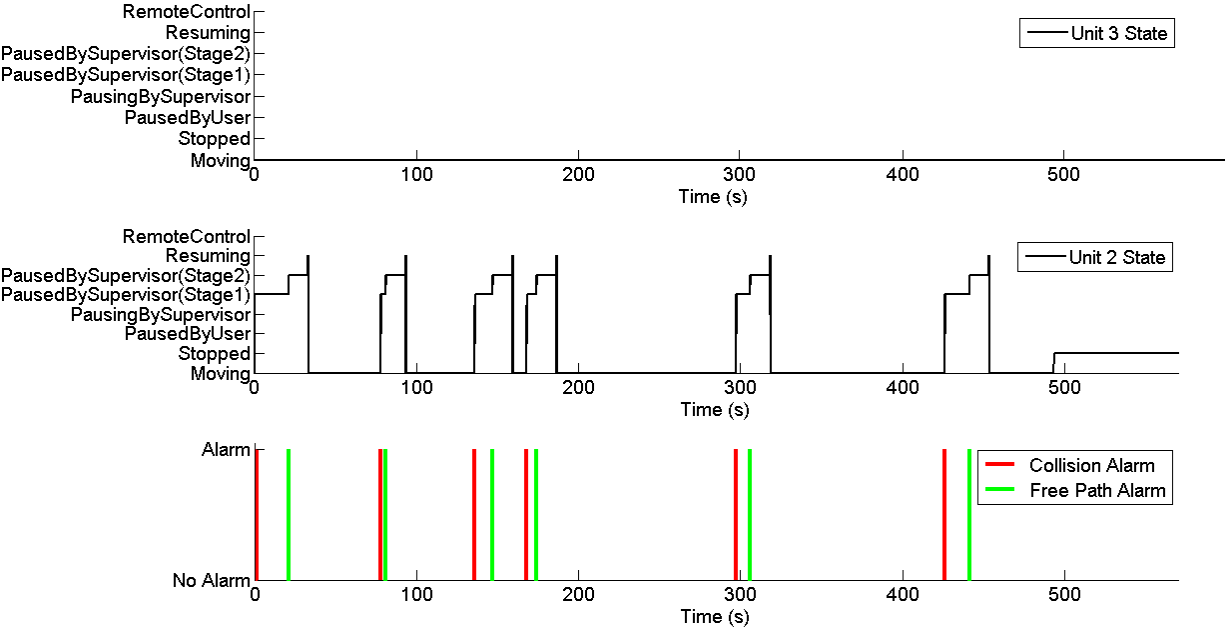
Collision supervisor outputs and unit states during the mission.

When the first collision was detected (red cross in Figure 14a), the alarm was propagated to the Fault Recovery Module, and it forced unit 2 to pause its mission. Unit 3 kept moving; once it moved a sufficient distance, the collision supervisor detected a free path for the unit 2 manoeuvre and issued a free path alarm. This alarm was processed by the Fault Recovery Module, and it restarted unit 2 (Figure 14b). According to the initial conditions, if the units kept moving at the same speeds, because they are following analogous trajectories, they should not coincide at any point later in the mission. However, due to mechanical reasons, unit 3 was considerably slower than the second unit during the turn manoeuvres. Thus, tractor 2 reached a new risky position (Figure 14c) and was paused again by the Fault Recovery Module. Unit 3 finally finished the turn manoeuvre and moved away, and unit 2 was then restarted (Figure 14d). In the second turn of unit 3 (Figure 14e–g), when the unit took so much time to drive around the curve, unit 2 had to be stopped twice (it was resumed because the collision supervisor calculated the future positions according to the expected speeds, and unit 3 in the expected location). For the next two turning manoeuvres, two new collisions were detected (Figure 14i,k). Consequently, unit 2 was paused; once unit 3 completed the manoeuvres and moved a sufficient distance, unit 2 was restarted (Figure 14h,j). Finally, both units finished their missions (Figure 14m,n). Figure 15 shows the internal states of the units during the mission as well as the outputs of the collision supervisor. The collisions and free path alarms issued by the collision supervisor are displayed in red and green lines, respectively. There are six red lines because six collisions were detected (Figure 14a,c,e,g,i,k), and there are six green lines because 6 collisions were prevented (Figure 14b,d,f, h,l).

Unit 3 maintained the same state during the entire mission because the Fault Recovery Module decided to pause/resume unit 2 every time. When the first collision was detected, the Fault Recovery Module could choose which unit to pause; at this time, both possibilities were available and unit 2 was randomly selected as all options were equally advantageous. However, during the remaining collision situations, the Fault Recovery Module selected unit 2 as no other option was available; the pausing of unit 3 did not prevent the collision.

Every time a collision was detected, the state of unit 2 changed quickly to the “PausingBySupervisor” state and remained there until the pause order was internally processed. It was then changed to the “PausedBySupervisor (Stage1)” state. In that state, the unit remained paused until the free path alarm was issued, and then, the unit supervisor state reached the “PausedBySupervisor (Stage2)” state. In that state, the unit remained paused again, but the supervisor started a countdown of 10 s before resuming operation of the unit. These 10 s, which were experimentally determined, were employed to enhance the safety conditions that prevent the unit from resuming when it remained in close proximity to the other vehicles (the supervisor can detect sufficient room with an accuracy of centimetres). After 10 s, operation of the unit was finally resumed, changing nearly immediately to the states “Resuming” and “Moving”.

A video that describes mission can be accessed in [40].

**Figure 16:**
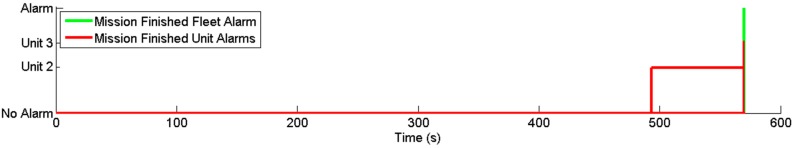
Fleet mission completed supervisor output (green signal) and inputs (red signals).

Finally, Figure 16 shows the results for the fleet mission completed by the supervisor. This supervisor receives as inputs the alarms generated by the remaining mission supervisors of the vehicles. Because both units completed the mission (red signals in the figure), the mission can be considered completed, and the supervisor issued the corresponding alarm (in green in the figure) as soon as it received the last mission completed signal from a vehicle.

## 6. Conclusions

The supervision system that was designed and developed was able to detect different failures, including dangerous situations, such as tractor out-of-track positions, inappropriate working speeds, incorrect states of the implements and potential collisions between units. The supervision system also detects important events, such as the completion of a trajectory and the end of the mission.

The distributed and multilevel approach is well suited to the distributed nature of the fleet of autonomous vehicles. The lowest level of supervision operates inside the units, taking care of the most urgent issues. A higher level is in charge of the more complex supervision that involves the entire fleet; this level is performed on an external computer that receives all of the information provided by the units (so it has a complete overview of the fleet status). The third level allows a human operator to monitor the underlying system and take control if needed. The external level is also divided into different sub-levels that contain different basic supervisors. The modular and hierarchical architecture has proven to be a useful framework for incrementally implementing complex supervisor systems based on lower-level and simpler supervisors. Thus, the supervision system could easily be extended to detect any type of failure just by adding new low-level supervisors.

The proposed system successfully performs the three main supervision functions: fault detection, fault diagnosis and fault recovery. It can complete these functions for any of its three main levels.

Two realistic tests based on real agricultural tasks were designed to test the system, and the supervisor succeeded in both tasks with high performance. Thus, the supervision system and architecture are highly useful for supervising fleets, particularly fleets of agricultural vehicles.

In future work, the supervisor could be improved by enhancing the fault recovery functionality, which could be accomplished by linking more alarms to the Fault Recovery Module to neutralise more failures. In addition, tests with larger fleets should be performed to test the scalability of the system. To date, a maximum of three vehicles have been used in real trials. A fleet with more units will provide the system with more information to process, and the processing can be a bottleneck because the system operates in real time. Finally, some simple constraints could be added to some of the supervisors to avoid alarms when they are not clearly needed. For example, the supervisor of the state of the implements could account for the delay in the response of the nozzles by allowing for some response time difference. Moreover, in some cases, the alarm may be activated/deactivated very often, such as when the speed supervisor has a small threshold. For these cases, it could be useful to add a filter in the Alarm Notification Manager so as not to overwhelm the operator with redundant information. The study of different development options is being planned to achieve a flexible policy system in which the policies can be adjusted during runtime, either manually by the operator or automatically, depending on the operator’s current workload.
